# Direct Laser Writing of SERS Hollow Fibers

**DOI:** 10.3390/nano12162843

**Published:** 2022-08-18

**Authors:** Jiajun Li, Yunyun Mu, Miao Liu, Xinping Zhang

**Affiliations:** Institute of Information Photonics Technology, Beijing University of Technology, Beijing 100124, China

**Keywords:** surface-enhanced Raman scattering, direct laser writing, inner wall, hollow fiber, sensors for flowing liquids

## Abstract

We report the direct laser writing (DLW) of surface-enhanced Raman scattering (SERS) structures on the inner wall of a hollow fiber. Colloidal gold–silver alloy nanoparticles (Au–Ag ANPs) are firstly coated onto the inner wall of a hollow fiber. A green laser beam is focused through the outer surface of the hollow fiber to interact with colloidal Au–Ag ANPs so that they become melted and aggregated on the surface of the inner wall with strong adhesion. Such randomly distributed plasmonic nanostructures with high density and small gaps favor the SERS detection of low-concentration molecules in liquids flowing through the hollow fiber. Such a SERS device also supplies a three-dimensional microcavity for the interaction between excitation laser and the target molecules. The DLW system consists mainly of the flexible connection between the motor shaft and the hollow fiber, the program-controlled translation of the hollow fiber along its symmetric axis and rotation about the axis, as well as the mechanical design and the computer control system. This DLW technique enables high production, high stability, high reproducibility, high precision, and a high-flexibility fabrication of the hollow fiber SERS device. The resultant microcavity SERS scheme enables the high-sensitivity detection of R6G molecules in ethanol with a concentration of 10^−7^ mol/L.

## 1. Introduction

Raman scattering spectroscopy [1,2] characteristically has high specificity for the detection of various molecules, and target molecules with fingerprinting spectroscopic responses can be recognized precisely. These features make Raman spectroscopy an excellent detection technique for environmental science, food safety, pharmaceutical and biological testing [3,4,5]. However, Raman signals are very weak and the enhancement of detection sensitivity is always important for Raman sensing techniques. Thus, surface-enhanced Raman scattering (SERS) spectroscopy is mostly employed in the trace detection of molecules in various forms. Localized surface plasmon resonance is the basic physics for SERS sensing, in which randomly [6,7] or periodically [8,9] distributed metallic nanostructures supply SERS hotspots [9,10,11,12] by the locally generated strong optical electric fields [6,7]. Therefore, the realization of high-quality SERS substrates is the first and most important precondition for SERS sensing techniques. Mesoporous metal films [13,14,15], nanoislands [16], nanocavities [17], and nanospheres [18] made of gold or silver on planar substrates have been widely employed in the fabrication of SERS substrates.

For the detection of low-concentration molecules in liquids, although the excitation volume extends in a depth of millimeter- or centimeter-scales, the effective interaction distance of a planar SERS substrate is only within 100 nm [19]. Thus, the SERS signal contributes only a very small portion to the collected Raman scattering spectrum. Moreover, the large scattering angles of the SERS signals over the hotspots on the metallic nanostructures result in low collection efficiency in far-field detection, where the detection distance is further extended by the design of the container of the liquid samples. These problems limit the application of SERS sensing techniques in the direct detection of low-concentration molecules in liquids.

Compared with conventional planar SERS substrates, hollow fibers with SERS [20,21,22] structures not only enable larger-area interactions, but also supply 3D microcavities for optical confinement and optical feedback mechanisms [23,24]. The closely arranged metallic nanostructures supply high-density SERS hotspots, as well as high reflection and a strong optical scattering of light. Therefore, the SERS-functionalized inner wall of the hollow fiber is also an efficient waveguide for the excitation laser and a flowing channel for the solution containing target molecules. Thus, SERS hollow fibers are promising for the in situ and real-time detection of low-concentration molecules in liquid samples [25,26,27]. However, the inner-wall SERS structures produced by thermal annealing have the disadvantages of relatively low homogeneity, less continuity, a low density of hotspots, low controllability, and low reproducibility [27].

In this work, we use a chemical method to synthesize gold–silver alloy nanoparticles (Au–Ag ANPs) and coat them on the inner wall of a hollow fiber. Using a laser annealing process, we are able to fabricate the SERS structures on the inner wall of the hollow fiber and the focused laser beam scans the wall surface by a program-controlled trace. Thus, a three-dimensional SERS microcavity is produced inside the hollow space. Using such a SERS microcavity, we successfully detect the R6G molecules in ethanol with a concentration as low as 10^−7^ mol/L and achieve a SERS enhancement factor larger than 10^5^. Therefore, the direct laser writing method provides an advantageous technique for the preparation of SERS structures in hollow-core fibers for the direct detection of low-concentration molecules in liquids.

## 2. Direct Laser Writing (DLW) of SERS Structures on the Inner Wall of a Hollow Fiber

### 2.1. Basic Principles for the DLW Technique

**Figure 1:**
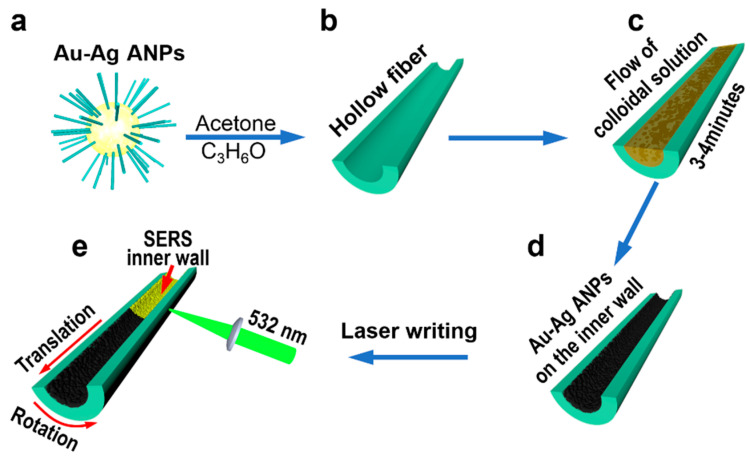
Preparation of SERS nanostructures on the inner wall of a hollow fiber. (**a**) Chemically synthesized gold and silver alloy nanoparticles (Au–Ag ANPs) with ligands. (**b**) Quartz hollow fiber. (**c**) Filling the hollow fiber with the colloidal solution of Au–Ag ANPs in acetone. (**d**) Coating of the colloidal Au–Ag ANPs onto the inner wall of a hollow fiber by flowing the colloidal solution for 3–4 min. (**e**) Direct laser writing of the SERS hollow fiber.

The Au–Ag ANPs were synthesized chemically using the recipe described in the section of the experimental methods. Figure 1 illustrates schematically the preparation of 3D SERS microcavity structures using the Au–Ag ANPs and the DLW technique. The produced powder of Au–Ag ANPs were suspended in acetone with a concentration of 100 mg/mL as a colloidal solution; the ANPs had a size of 3–8 nm and were covered with ligands for good dispersion in the colloidal solution, as shown in Figure 1a. The as prepared colloidal solution was filtrated three times using a butterfly filter to remove the bulky impurities. The hollow fiber was supplied by Beijing Scitlion Technology Corp., Ltd. and it was made of quartz glass with a refractive index of 1.461 at 532 nm and 1.454 at 785 nm. In the cleaning process of the hollow fiber, it was first submerged in anhydrous ethanol with ultrasonic cleaning for 5 min; then, it was submerged in deionized water for ultrasonic cleaning for 5 min. After replacing the deionized water, the hollow fiber was cleaned again ultrasonically for 5 min. Finally, the cleaned hollow fiber was dried in a drying cabinet before use. After the hollow fiber was thoroughly cleaned, the surface of the inner wall was modified with 5% aqueous sodium hydroxide solution, as shown in Figure 1b. Then, the Au–Ag ANPs colloidal solution was injected into the hollow fiber and was made to flow through the hollow with a diameter of 600 µm and a length of 5 cm for 3–4 min, as shown in Figure 1c. After the rapid evaporation of acetone, a layer of Au–Ag ANPs was left on the inner wall of the hollow fiber, as shown in Figure 1d.

The laser annealing process was performed in the step shown in Figure 1e. A continuous-wave laser beam at 532 nm with a power of 760 mW was focused by a lens with a focal length of 100 mm onto the Au–Ag ANPs coating the inner wall of the hollow fiber from the outer surface. The choice of a 532 nm laser as the writing laser source was based on the consideration that the green laser wavelength at 532 was very close to the peak wavelength of the absorption spectrum, which is supplied in Figure S1, and a peak wavelength of 504 nm could be identified in the absorption spectrum of the thin film spin-coated by the colloidal solution of the Au–Ag ANPs in acetone. The laser spot was measured to have a diameter of about 160 µm on the inner wall, corresponding to a laser intensity of 3.7 W/cm^2^. The laser-annealing process enables the sublimation of the ligands, the melting and aggregation of the Au–Ag ANPs, and the formation of the matrix of high-density Au–Ag alloyed nanoislands on the curved surface of the inner wall. The ligands were produced to cover the ANPs during the synthesis process, and are important for dispersing the ANPs in the colloidal solution. By adjusting the laser power, focusing area, translation and rotation speeds, the size and density of the Au–Ag ANPs can be optimized. Thus, a cylindrical SERS microcavity forms in the curved hollow space. The most important advantage of such Au–Ag ANPs lies in the combination of the better stability of Au nanoparticles with the better SERS performance of the silver nanoparticles. Related investigations and analysis can be found in our previous work in [28]. Our experimental results in Figure S2 supply strong evidence for the technical design and the proposed mechanisms. As shown in the left panel of Figure S2 by a photograph of the sample and an optical microscope image of a segment of the hollow fiber, the colloidal Au–Ag ANPs cover the inner surface of the hollow fiber completely and are homogeneous without any obvious discontinuity or defects. The right panel of Figure S2 is the SEM image of the fabricated SERS inner wall after the sample is annealed by the writing laser beam, where excellent alloyed nanostructures can be observed.

### 2.2. Program-Controlled DLW System

**Figure 2:**
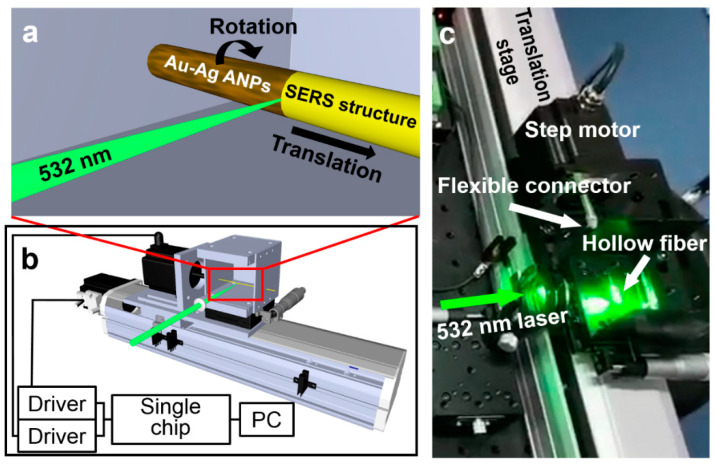
(**a**) Schematic illustration of the principles for direct writing of the SERS hollow fiber. (**b**) The conceptual drawing of the construction of the direct laser writing system. (**c**) Photograph of the home-built DLW system.

**Figure 3:**
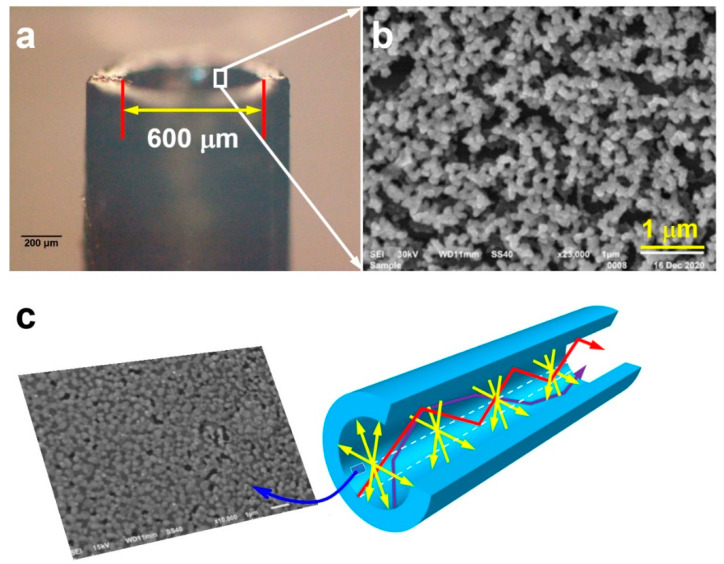
(**a**) Optical microscopic image of the SERS hollow fiber. (**b**) The SEM image of a local area on the inner wall of the SERS hollow fiber. (**c**) Schematic illustration of the principles for the SERS microcavity.

Figure 2a,b show the schematic design of the DLW system and Figure 3c shows a photograph of the home-built system. The hollow fiber is mounted on the combined system of a linear translation stage and a rotation stage via a flexible connection. The mechanical design ensures well-controlled rotation and smooth translation along the axis of the hollow fiber. The laser beam is fixed in its orientation and focusing position and the hollow fiber is rotated while being moved forward to accomplish the scanning of the interaction between the laser focusing spot with the ANPs on the inner wall. This scanning process is controlled by computer programs via two step motors, where the hollow fiber is rotated by a full circle before being moved forward by a step. One of the step motors is responsible for forward moving and the other for rotating the hollow fiber. It needs to be noted that the length scale for each step for moving forward is smaller than the diameter of the focused laser spot so that the scanning process may cover the whole area of the inner wall surface.

The diameter of the laser spot on the focus is measured to be about 160 µm, where the knife-edge method is employed. The linear forward-moving motion is in steps of about 135 μm; thus, no gaps will be left between the two adjacent full scanning circles. The linear translation in the axial direction has a resolution of 1 µm and the rotation about the axis has an accuracy of 0.036°, which are sufficient to ensure smooth and homogeneous interaction over the whole scanning area. The rotation step motor should also rotate at a speed that ensures no-gap scanning of the whole surface of the inner wall of the hollow fiber, which is determined by considering the size of the laser spot, the dwell time on each writing site, and the whole circumference of the inner surface of the hollow fiber. Therefore, the angular velocity was set to about 120°/s. In fact, such a DLW system can be utilized to achieve various configurations of metallic ANP structures on the circular inner wall. Separate program-controlled linear and circular movements facilitate the flexible design and writing of the SERS structures.

## 3. 3D SERS Microcavity by the Curved Inner Wall of the Hollow Fiber

Figure 3a shows the optical microscopic image of the SERS hollow fiber on one end, where the 5 cm long hollow fiber has been cut into two segments in the middle so that the microscopic images reflect the typical features in the mid-section. The inner diameter of the hollow fiber is 600 µm. Figure 3b shows the SEM image of the Au–Ag ANPs on the inner wall of the hollow fiber. Clearly, Au–Ag ANPs with a mean diameter of about 100 nm are stacked randomly into a 3D matrix, as shown by the diagram in Figure S3, which is a statistic evaluation on the mean diameter of the direct-laser-written Au–Ag ANPs on the inner wall of the hollow fiber using the SEM image in Figure 3b. Basically, the sizes of the alloyed nanoparticles are distributed in a range from 80 to 120 nm, so that a mean diameter of about 100 nm can be clearly justified. Although relatively larger irregular holes are distributed between the dense aggregates, very small gaps can be justified between the Au–Ag ANPs. The holes supply space and mechanisms for the infiltration of the molecule into the 3D structures, whereas the small gaps supply high-density hotspots for SERS detection. Therefore, the Au–Ag ANPs are well configured into microscopic structures to favor SERS sensing. It also needs to be noted that although the Au–Ag ANPs are randomly distributed with gaps and holes modulating the surface smoothness, the close aggregation of the ANPs with high density produces a stable and continuous metallic surface with high reflection and strong optical scattering. This lays the basis for the formation of 3D microcavity schemes in the hollow space. We need to stress here that although we have optimized some of the parameters, we did not reach an optimal SERS surface to be evaluated by only one or two indices. We still were able to improve the surface properties to obtain the best SERS performance, instead of only the structural properties.

The SEM images in Figure 3b,c verify the successful fabrication of the plasmonic structures consisting of Ag–Au ANPs coated on the inner wall of the hollow fiber. Since the position of this microscopic characterization is randomly selected in the mid-section of the hollow fiber, the demonstrated homogeneous structures are characteristic of the whole hollow fiber, where the alloyed nanoparticles are uniformly and continuously distributed on the inner wall. Nevertheless, we need to understand that on both ends of the hollow fiber, the size and distribution of the ANPs are somewhat different, which is influenced by different solvent evaporation and ANP-annealing effects on both ends from the mid-region of the hollow fiber. Due to the high optical reflection and strong plasmonic optical scattering, the hollow fiber still functions as a waveguide for the inside propagating light beam. According to our verification test, there was less than a 30% loss of laser energy for the input on one end and the output on the other, where we coupled a 785 nm laser beam into the hollow fiber. This energy loss can be reduced further when the hollow fiber is filled with liquids, e.g., ethanol or water, due to the better interfacial conditions, which helps the excitation laser fully interact with the sample molecules to improve the SERS efficiency. The complex nanogaps and nanopores formed by the close arrangements of the Au–Ag ANPs facilitate the formation of SERS hotspots and the adsorption of the sample molecules near them. Actually, the size and density of the produced ANPs, as well as the gaps between them, can be adjusted either by using different lasing processing parameters or by using different conditions for the coating with colloidal alloy nanoparticles. Using different laser powers, different exposure times, different concentrations of the colloidal solution, and different times for flowing the colloidal solution through the hollow fibers are possible approaches.

Figure 3c schematically shows, in the right panel, the basic principles for the formation of a 3D microcavity inside the fiber hollow. The following mechanisms are responsible for the strongly enhanced interaction between the excitation laser and the target molecules. (1) The small space in the fiber hollow confines all of the excitation laser energy into the cylinder with a diameter of 600 µm, implying much enhanced excitation intensity and much enlarged excitation volume in the whole space of the hollow fiber as long as 5 cm [29]. (2) The curved surface of the cylindrical inner wall with high optical reflection supplies further refocusing mechanisms for the propagating laser beam in the hollow fiber. (3) Optical feedback for the excitation laser through reflection and scattering by the curved metal surface in the cross-sectional plane, as illustrated by the yellow arrows. (4) Multifold optical reflection as a waveguiding mechanism in the meridian plane multiplies the excitation of the SERS hotspots, as indicated by the red arrow. (5) The random propagation of the excitation laser along the SERS surface is established by the plasmonic scattering of the Au–Ag ANPs, as indicated by the zigzag arrow in purple. The SEM image in Figure 3c on the left panel shows the practical microscopic structures of the Au–Ag ANPs on the inner wall of the hollow fiber.

## 4. Sensing of Low-Concentration R6G Molecules in Ethanol

**Figure 4:**
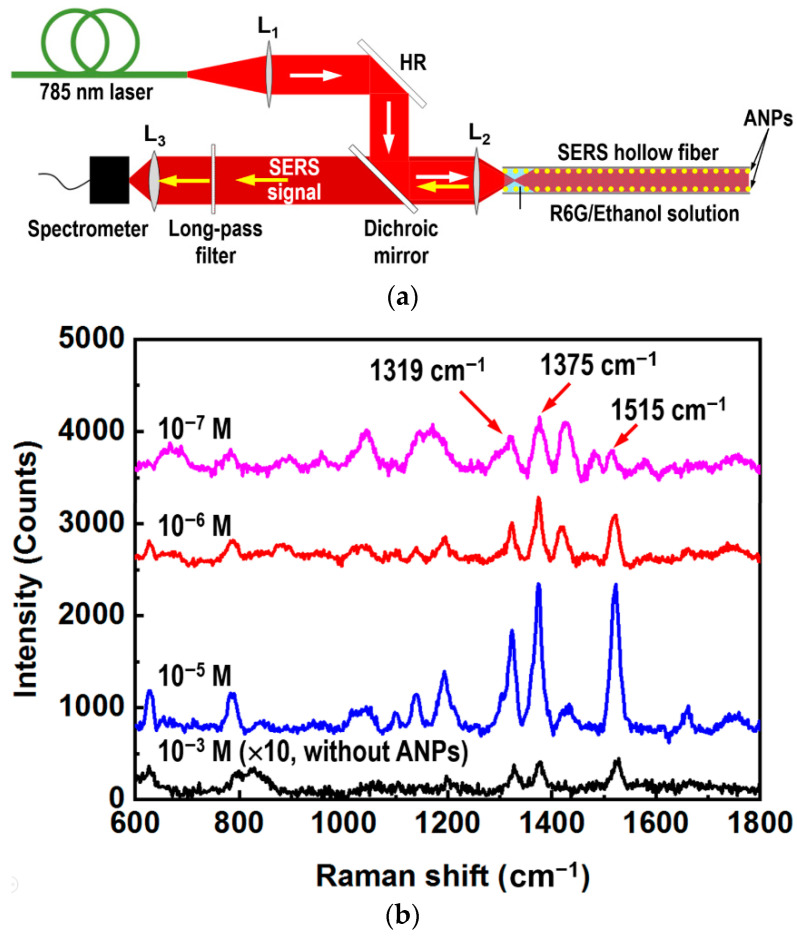
(**a**) Schematic illustration of the SERS detection system. (**b**) SERS signal spectra measured at different concentrations of the R6G/ethanol solution: 10^−3^ M (black), 10^−5^ M (blue), 10^−6^ M (red), and 10^−7^ M (magenta).

Figure 4 shows a schematic illustration of the experimental setup for SERS detection. A fiber-coupled 785 nm laser is used as the excitation (white arrow), which is recollimated by a lens (L_1_), reflected by a high-reflection mirror (HR) to a dichroic mirror, and focused by a lens (L_2_) into the SERS hollow fiber to excited the target molecules in the liquid filling the hollow space. The hollow fiber is filled with the R6G/ethanol solution with different concentrations. In sampling the target solution, the hollow fiber was dipped by one end into the R6G/ethanol solution, so that the solution was drawn into the hollow by the capillary force. Then, the hollow fiber was sealed on the other end as soon as the hollow was fully filled with the solution. Finally, the filled fiber was fixed in the Raman detection optical path. The back-scattering SERS signal beam (yellow arrow) was recollimated by L_2_ before passing through the dichroic mirror and a long-pass filter, so that the remaining excitation laser in the signal beam stopped completely. Therefore, only the SERS signal beam was collected by a lens (L_3_) and focused into the detection head of a fiber-coupled Raman spectrometer.

As a conventional characterization of the SERS sensing devices, we carried out Raman measurements directly on the R6G/ethanol solutions with different concentrations using the method demonstrated in Figure 4a. Figure 4b shows the measurement results where three concentrations of 10^−5^, 10^−6^, and 10^−7^ M were employed for the R6G/ethanol solutions. The 785 nm laser with a total power of 200 mW was used as the excitation and an integration time of 1 s was used for all Raman spectroscopic measurements. Although multiple Raman peaks can be observed in Figure 4b, the most typical signals were located at 1319, 1375, 1515, and 1660 cm^−1^, corresponding to the stretching vibration modes of the C-C bonds on the benzene ring. For comparison, we also supply the Raman signal measured on the solution with a concentration of 10^−3^ M, where the solution was injected into a hollow fiber without the SERS inner wall and the Raman spectrum was enlarged by a factor of 10.

The SERS enhancement factor can be calculated by:

E = (I_SERS_ × C_RS_)/(I_RS_ × C_SERS_), (1)

where I_SERS_ and I_RS_ are the peak intensities of the Raman signals measured using a SERS hollow fiber and a blank one without the ANP coating, and C_SERS_ and C_RS_ are the corresponding concentrations of the sample solutions, respectively. Using Equation (1) and the measurement data in Figure 4b, we can calculate the SERS enhancement factors for the signals at 1319 cm^−1^, 1375 cm^−1^ and 1515 cm^−1^. The calculation results for these enhancement factors are E_1319_ = 1.35 × 10^4^, E_1375_ = 1.37 × 10^4^, and E_1515_ = 5.86 × 10^3^, which are all in the order of 10^4^. If considering the enhancement of the Raman signal by the confinement of the excitation laser by the hollow fiber, there is a further enhancement factor larger than one order. Therefore, we can evaluate a SERS enhancement factor as large as 10^5^. In fact, without the confinement by the hollow fiber, we were not able to measure Raman signals through the direct detection of R6G/ethanol with a concentration of 10^−3^ M.

## 5. Conclusions

We have demonstrated the direct laser writing of a SERS sensing device consisting of Au–Ag ANPs on the inner wall of a hollow fiber. The alloying of silver with gold in the plasmonic nanoparticles combines the advantages of the stability of gold and the excellent SERS performance of silver. High-quality plasmonic nanostructures were produced with the homogeneous and continuous distribution of the gold–silver alloy nanoparticles on the inner-wall surface of a hollow fiber with a diameter of 600 µm and a length of 5 cm. Such a structure with high reflection and strong optical scattering favored the formation of a 3D SERS microcavity, facilitating multifold optical confinement and optical feedback mechanisms, enhancing further the SERS effect. An enhancement factor in the order of 10^5^ was achieved. This technique not only provides a direct laser annealing technique that is more advantageous than conventional thermal annealing, but also supplies an efficient method for the direct detection of low-concentration molecules in liquids.

## 6. Experimental Methods

### 6.1. Synthesis of Au–Ag Alloy Nanoparticles (Au–Ag ANPs)

The gold and silver alloy nanoparticles used in this paper were synthesized chemically using the follows procedures:

(1) First, 1.143 g tetrabutylammonium bromide (TOAB) was added into 60 mL toluene to fully dissolve; then, 2.44 mL aqueous sulfuric acid solution with a concentration of 1.5 mol/L was added to the solution;
(2) Next, 0.173 g silver nitrate was added into the above-prepared solution with sufficient stirring, until the solution became clarified;
(3) Then, 0.11 g H[AuCl₄]·4H₂O was added into the above-prepared solution. Thus, the water-insoluble TOAB formed ionic conjugates with Au^3+^ and Ag^+^, which were transferred to the toluene phase, preventing Ag^+^ and [AuCl_4_]^-^ from forming AgCl and Au(OH)_3_ precipitate in the aqueous phase. The toluene phase became red–brown in color;
(4) After the aqueous phase was clarified and transparent, it was separated and the organic phase was retained. Then, 0.371 g hexanethiol was added to the solution of the organic phase before the solution was heated to 40 °C in a water bath;
(5) Next, 15 mL aqueous sodium borohydride solution with a mass concentration of 1.8% was added to the solution prepared in (4), and was heated and stirred for 12 h. Thus, hexanethiol-protected gold–silver alloy nanoparticle colloids were prepared;
(6) The aqueous phase was separated and the organic phase was distilled under reduced pressure at 50 °C in water bath, so that a black oily viscous liquid was obtained;
(7) The product in (6) was then washed with methanol under centrifugation. The washing of the black precipitate was repeated 4 to 5 times. Gold and silver alloy nanoparticles were thus produced after being dried by blowing with nitrogen. The transmission electron microscope (TEM) image and the energy-dispersion X-ray spectrum (EDS) measured on the synthesized Ag–Au ANPs are given in Figure S4 and Figure S5, respectively. The TEM image in Figure S4 shows a diameter of roughly 3–8 nm for the Ag–Au ANPs and the EDS data determine a weight ratio of 3:5 or an atomic ratio of roughly 1:1 between the Ag and Au elements. The photographs of the as-prepared powder sample of the ANPs and the colloidal solution of the ANPs in acetone with a concentration of 100 mg/mL are shown in Figure S6.

### 6.2. Microscopic and Spectroscopic Measurements

The SEM images of the hollow fiber were taken with a Nikon’s Eclipse LV100ND optical microscope, and the surface morphology of inner wall was characterized by JSM6510 of JEOL. Raman detection was completed through a self-built optical path, in which the Raman excitation light source was Laser785-0.08-500-FC-3A of Oceanhood Optoelectronics (Shanghai, China). Raman signals were obtained by QE pro of Ocean Optics (830 Douglas Ave. Dunedin, FL 34698, USA).

### 6.3. The Optical and Electromechanical Design

The laser source for the LDW was a 532 nm laser (LWGL532-083683, purchased from Beijing Laserwave Optoelectronics Technology Co., Ltd., Beijing, China) with a maximum output power of more than 1 W; however, about 760 mW was utilized in the LDW process. The laser power was measured before the focusing lens using a power meter THORLABS PM100D with a detection head THORLABS S350C.

The direct output laser beam was focused by a plano-convex lens with a focal length of 100 mm to produce a laser spot with a diameter of about 160 µm on the focus. The corresponding laser intensity was about 3.7 W/cm^2^. The LDW system was built up by step motors (AM23HS2450-03 with a driver of SRX08-S from MOONS), the precision slide module was SH-L10-S200-BC of SHINING OE, the lower computer was Arduino UNO of Arduino, and the control program was independently developed.

## Supplementary Materials

The following supporting information can be downloaded at: https://www.mdpi.com/article/10.3390/nano12162843/s1, Figure S1: Absorption spectrum measured on the spin-coated thin film of colloidal Au-Ag-ANPs on a glass substrate; Figure S2: Photographs (top-left) and optical microscope (bottom-left) images of the hollow fiber with its inner wall coated with colloidal Au-Ag-ANPs, as well as the SEM image (right) of a local area after being annealed by the writing laser beam; Figure S3: Statistic evaluation on the mean diameters of the direct-laser-written Au-Ag-ANPs on the inner wall of the hollow fiber using the SEM image in Figure 3b; Figure S4: TEM image of the Au-Ag-ANPs; Figure S5: EDS measurement on the Au-Ag-ANPs; Figure S6: Photographs of the synthesized Au-Ag-ANPs in powders (left) and colloidal solutions in acetone (right).
