# Supplementary material for: Direct Laser Writing of SERS Hollow Fibers
Source: Nanomaterials (Basel). 2022 Aug 18;12(16):2843. doi: 10.3390/nano12162843 (PMC9413988; doi:10.3390/nano12162843)
Supplement: Supplementary file 1 [file nanomaterials-12-02843-s001.zip › nanomaterials-1827253-supplementary.pdf]

Supporting information

Direct Laser Writing of SERS Hollow Fibers

Jiajun Li, Yunyun Mu, Miao Liu and Xinping Zhang *

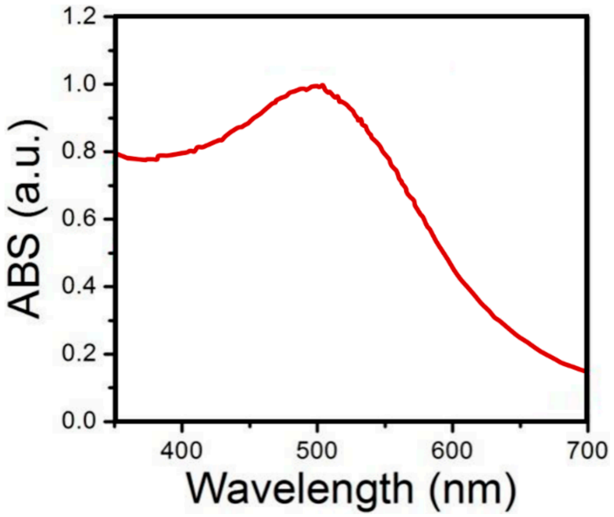

Figure S1 Absorption spectrum measured on the spin-coated thin film of colloidal Au-Ag-ANPs on a glass substrate.

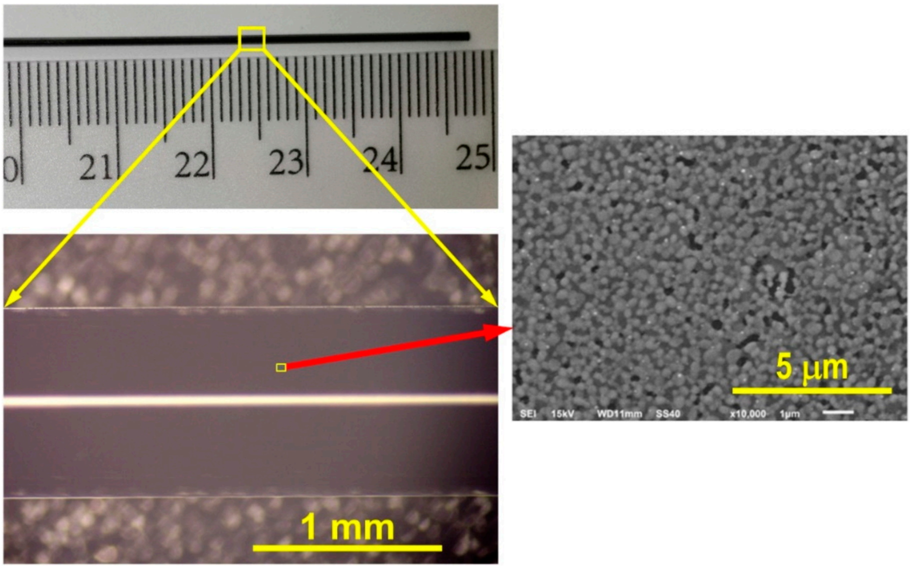

Figure S2 Photographs (top-left) and optical microscope (bottom-left) images of the hollow fiber with its inner wall coated with colloidal Au-Ag-ANPs, as well as the SEM image (right) of a local area after being annealed by the writing laser beam.

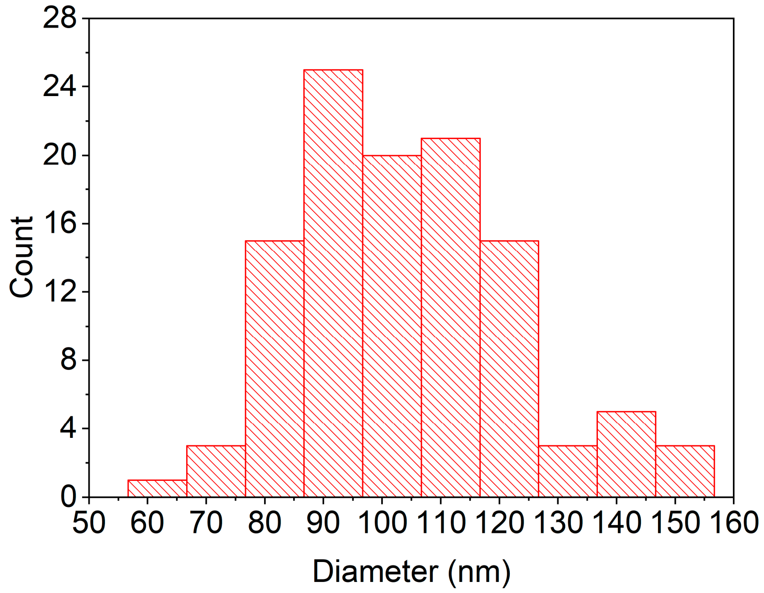

Figure S3 Statistic evaluation on the mean diameters of the direct-laser-written Au-Ag-ANPs on the inner wall of the hollow fiber using the SEM image in Fig. 3(b).

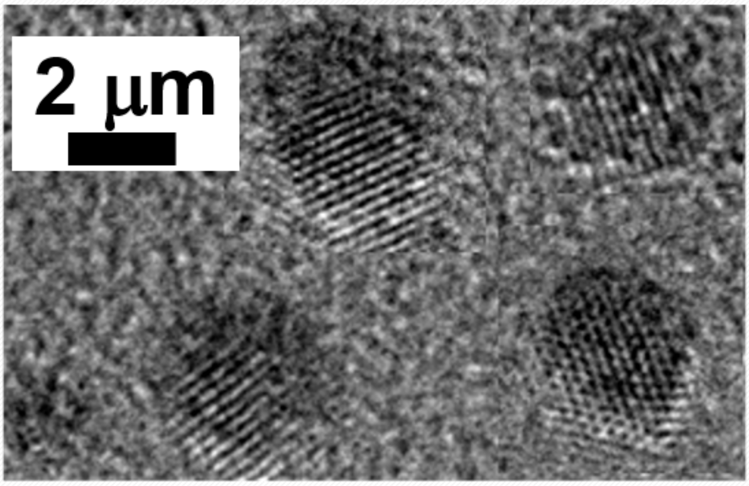

Figure S4 TEM image of the Au-Ag-ANPs.

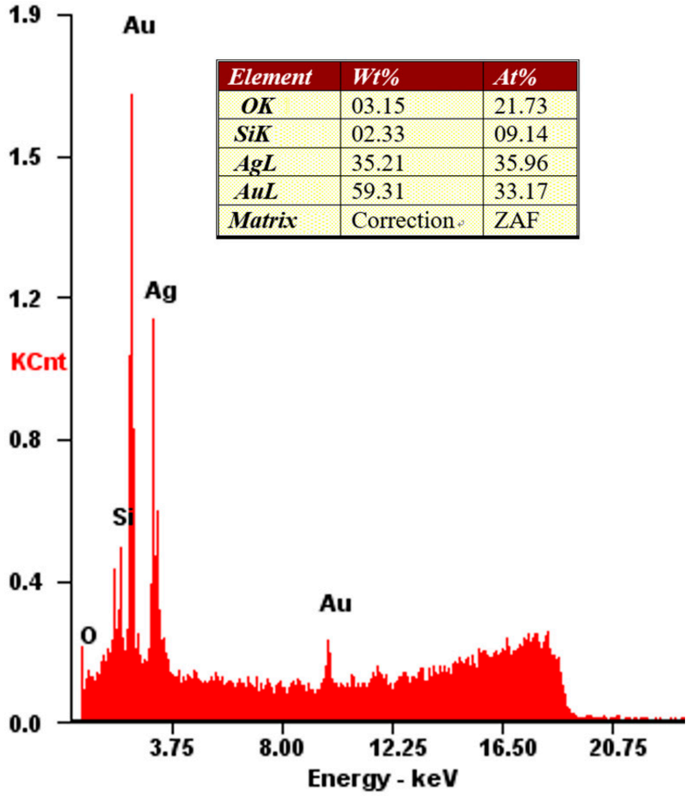

Figure S5 EDS measurement on the Au-Ag-ANPs.

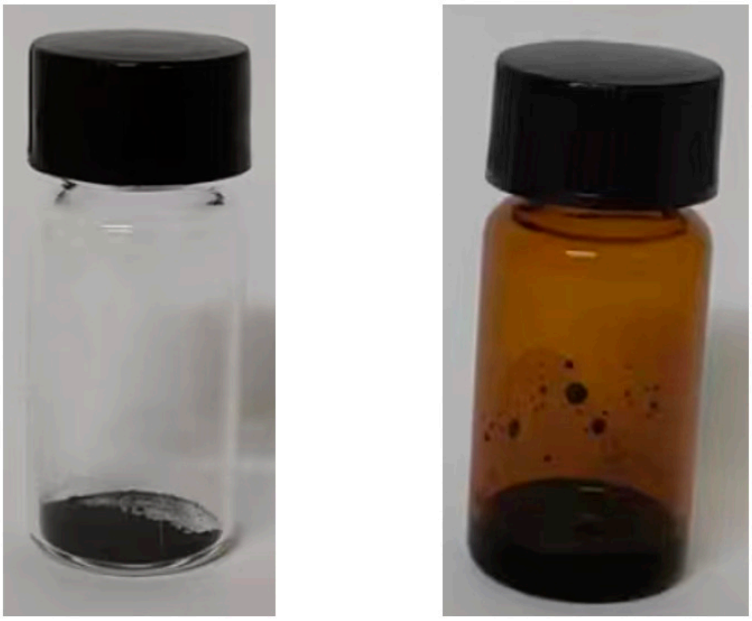

Figure S6 Photographs of the synthesized Au-Ag-ANPs in powders (left) and colloidal solutions in acetone (right).
